# In vitro activity of β-lactamase inhibitors avibactam, relebactam and vaborbactam in combination with aztreonam against metallo-β-lactamase producing *Escherichia coli* clinical isolates

**DOI:** 10.1007/s10096-026-05538-w

**Published:** 2026-06-23

**Authors:** Clément Viguier, Samanta Freire, Maxime Bouvier, Christophe Le Terrier, Laurent Poirel, NARA Network, A. Westers, M. Imperiali, L. Pozzi, D. Balzari, G. Vaninetti, C. Cirillo, V. Gaia, E. Pianezzi, G. Martinetti Lucchini, F. Baggi Menozzi, A. Jayol, C. Guyon, D. Hyden, M. Maitrejean, V. Deggi-Messmer, D. Bandeira, C. Fournier, S. Pfister, C. Nusbaumer, L. Bertaiola Monnerat, J. Schrenzel, G. Renzi, A. Cherkaoui, D. Andrey, S. Emonet, M. Eyer, R. Maret, A. Belo, D. Mabillard, M. Moraz, K. Herzog, V. Gisler, M. Oberle, C. Castelberg, H. Fankhauser, S. Graf, N. Dubey, C. Guler, M. Schoenenberger, U. Karrer, F. Piran, C. Andreutti, M. Dessauges, T. Schmid, B. Suterbuser, I. Mitrovic, V. Bruderer, P. Staehli, B. Schnell, C. O. Marti, I. Steffen, A. Imhof, B. Preiswerk, V. Dilorenzo, C. Payen, D. Boschung, L. Comte, M. Schacher, M. Brandenberger, C. Zowa, C. Zehnder, M. Mathis, L. Basilico, G. Togni, P. Minkova, Y. Born, M. Kuegler, V. Povolo, S. Droz, M. Elzi, C. Casanova, C. Lang, A. Blaich, S. Schmid, B. Ivan, S. Mancini, O. Dubois, K. Narr, S. Schoch, S. Ellenberger, S. Seiffert, Patrice Nordmann

**Affiliations:** 1 Emerging Antibiotic Resistance Unit, Department of Medicine, Medical and Molecular Microbiology, Faculty of Science and Medicine, University of Fribourg, Chemin du Musée 18, CH-1700 Fribourg, Switzerland; 2 Department of Infectious and Tropical Diseases, Toulouse University Hospital, Toulouse, France; 3 NEO-I3D Research Group, Toulouse University Hospital, Toulouse, France; 4 Swiss National Reference Center for Emerging Antibiotic Resistance, Fribourg, Switzerland; 5 Division of Intensive Care Unit, University Hospitals of Geneva, Geneva, Switzerland

**Keywords:** Metallo-beta-lactamase, Relebactam, Vaborbactam, Aztreonam-avibactam, β‑lactam/β‑lactamase inhibitor combinations

## Abstract

Aztreonam-avibactam (ATM-AVI) is one of the few remaining therapeutic options for infections caused by metallo-β-lactamase (MBL)-producing Enterobacterales, yet resistance is increasingly reported. Alternative strategies are therefore critically needed. We investigated the in vitro activity of aztreonam in combination with three marketed β-lactamase inhibitors (avibactam, vaborbactam, relebactam) against 48 well-characterized ATM-resistant MBL-producing *Escherichia coli*, including 34 ATM-AVI-resistant isolates. Among ATM-AVI-susceptible isolates, ATM-relebactam and ATM-vaborbactam showed reduced activity compared with ATM-AVI (MIC_50/90_ 1/4 mg/L), with MIC_50/90_ values of 8/64 mg/L and 64/256 mg/L, respectively. Among ATM-AVI-resistant isolates, none of the combinations restored aztreonam susceptibility. CMY-type enzymes seem to play a pivotal role in the failure of both relebactam and vaborbactam to restore aztreonam susceptibility in our collection of isolates.

## Introduction

The aztreonam-avibactam (ATM-AVI) combination, which pairs the property of aztreonam (ATM) not to be hydrolyzed by metallo-beta-lactamases (MBLs) with the ability of avibactam (AVI) to inhibit most of the co-produced serine-β-lactamases (often mediating ATM resistance), represents one of the few therapeutic options for MBL-producing Enterobacterales [1]. Although relatively recently introduced into clinical practice, ATM-AVI resistance has already been reported worldwide in Enterobacterales isolates producing either MBLs or class D carbapenemases (OXA-48-type) [2]. This raises significant concerns, since MBLs and class D carbapenemases are frequently identified in Europe [3]. Consequently, the emergence of ATM-AVI resistance in carbapenem-resistant *Escherichia coli*, often linked with community spread, may limit the application of this novel combination as a last-resort therapy. ATM-AVI resistance in *E. coli* primarily stems from specific mutations or insertions within the penicillin-binding protein PBP3 encoding gene, associated with the production of plasmid-encoded CMY-type-β-lactamases, especially when co-produced with NDM-type MBLs [2, 4]. This resistance pattern has been predominantly observed among NDM-5-producing *E.coli* isolates [5]. Therefore, new therapeutic alternatives must be considered for infections caused by MBL-producing Enterobacterales.

The latest marketed β-lactamase inhibitors (BLIs) include avibactam, relebactam, and vaborbactam. Avibactam and relebactam, both diazabicyclooctane derivatives, demonstrated efficient inhibition of Ambler class A enzymes, including KPC, and moderate inhibition of Ambler class C enzymes. Considering class D enzymes, avibactam demonstrated a more potent inhibition activity on OXA-48-like enzymes than relebactam. Vaborbactam, a boronic acid derivative, has a similar spectrum of inhibition, with the notable exception of class D enzymes. All three inhibitors are ineffective against MBLs [6].

To exploit ATM’s unique properties, recent studies have assessed its in vitro efficacy in combination with these three BLIs against MBL-producing Enterobacterales. While strong evidence exists for the efficacy of ATM-AVI, data on ATM combined with relebactam or vaborbactam remain limited. The few in vitro data regarding MBL-producing isolates support aztreonam-relebactam (ATM-REL) as a useful option, with susceptibility rates up to 61.9%, though less effective than ATM–AVI [7–10]. Conversely, the in vitro efficacy of aztreonam-vaborbactam (ATM-VAB) remains controversial, with higher susceptibility rates than those obtained for the ATM-REL combination as reported by Emeraud et al., while vaborbactam demonstrated a limited effect on ATM susceptibility in another recent study conducted by Tao et al. [7, 9]. While most of the above-mentioned studies support the potential usefulness of relebactam, and to a lesser extent vaborbactam for MBL-producing Enterobacterales-related infections, data on ATM-AVI-resistant strains remain scarce. Clinical experience with these combinations is also extremely limited: only a few case reports have described the use of ATM combined with imipenem-relebactam or meropenem-vaborbactam to treat infections caused by MBL-producing Enterobacterales, most often with favorable outcomes, although no international guidelines currently recommend such combinations [1, 11–13].

Our study aimed to evaluate the in vitro susceptibility of aztreonam combined with three already marketed BLIs, namely avibactam, vaborbactam, and relebactam, against ATM-resistant MBL-producing *E. coli* isolates, with an emphasis on ATM-AVI-resistant strains.

## Materials and methods

### Bacterial isolates

A collection of 48 non-duplicate ATM-resistant MBL-producing *E. coli* clinical isolates that had been collected either by the Swiss National Reference Center for Emerging Antibiotic Resistance (NARA) but also through different international collaborations was used in the study. Those isolates originated from various clinical sources and geographic regions, and were previously characterized for their β-lactamase content and PBP3 sequences (Table 1). They produced the main representative MBLs, namely NDM-like (*n* = 41), VIM-like (*n* = 5) and IMP-like (*n* = 2) enzymes. Most of them co-produced other β-lactamases, mainly represented by CMY-like (*n* = 36), CTX-M-like (*n* = 17), TEM-like (*n* = 20), OXA-like (*n* = 12), or SHV-like (*n* = 1) enzymes. Among the tested isolates, 42 exhibited a 4-amino-acid insertion within their PBP3 (YRIN, YRIK or YRIP), known as a source of reduced susceptibility to ATM [4]. Thirty-four were categorized as ATM-AVI-resistant, based on the 2025 EUCAST breakpoint (> 4 mg/L) [14].

**Table 1:**
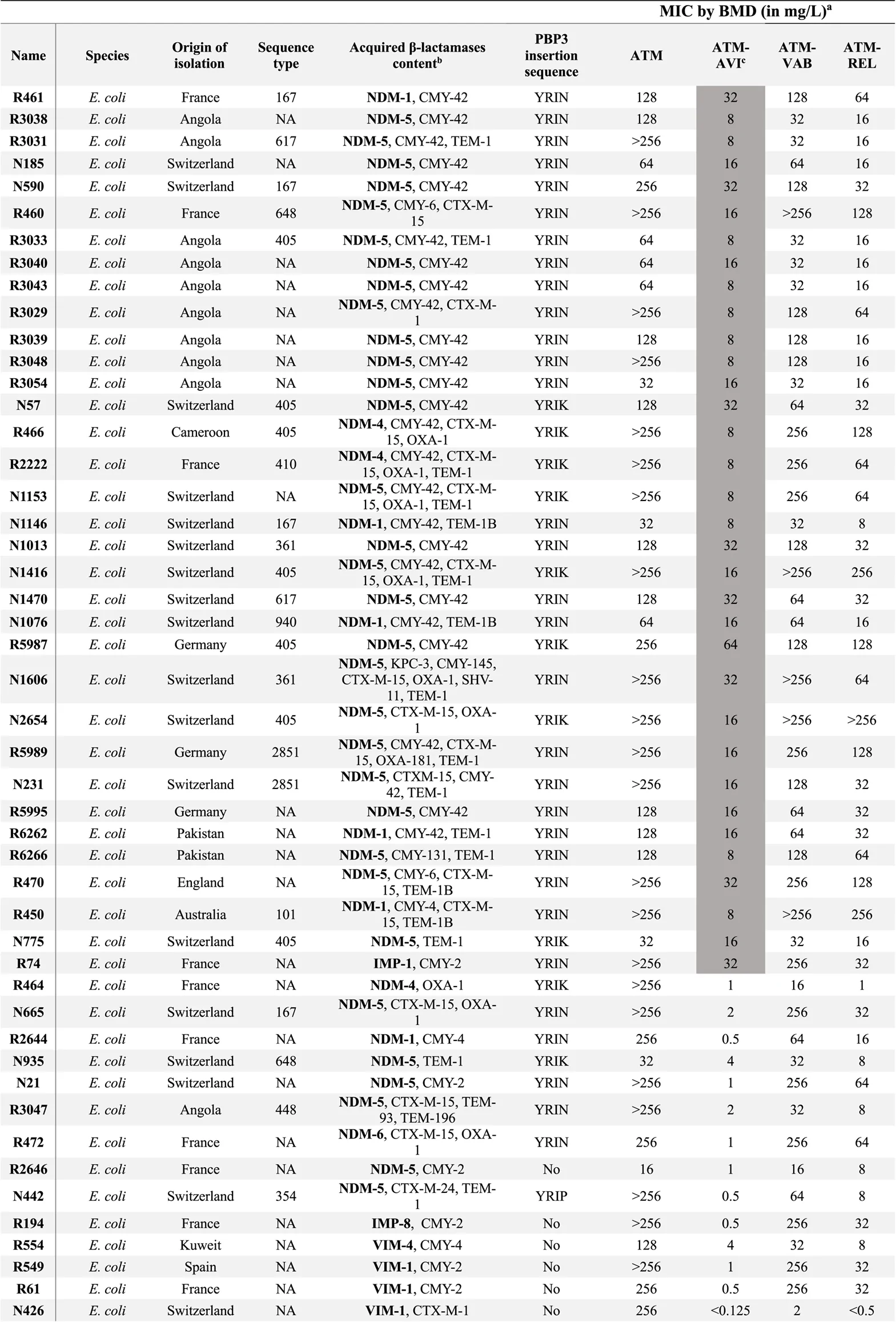
MICs of aztreonam (mg/L) alone and in combination with avibactam, vaborbactam and relebactam, using the BMD method

NA, non-available

^a^ Abbreviations: *ATM* aztreonam; *AVI* avibactam; *VAB* vaborbactam; *REL* relebactam

^b^ MBL enzymes are boldened

^c^ Dark grey colored MICs correspond to AZA-resistant isolates

The reference strains *E. coli *ATCC 25922, *K. pneumoniae* ATCC 700603 and ATCC BAA-2814 were used as quality control for all testing. All experiments were performed in triplicate

### Susceptibility testing

Minimum inhibitory concentrations (MICs) values were determined in triplicate by broth microdilution method, following EUCAST guidelines [15]. MICs were determined for ATM alone or in combination with fixed concentrations of avibactam (4 mg/L), relebactam (4 mg/L) or vaborbactam (8 mg/L). Resistance to ATM-VAB and ATM-REL was defined according to ATM breakpoint (> 4 mg/L for Enterobacterales) [14]. MIC_50_ and MIC_90_ values were calculated. *E. coli* ATCC 25922, *K. pneumoniae* ATCC 700603 and ATCC BAA-2814 served as the quality control strains for all testing.

## Results

Forty-eight non-duplicate MBL-producing *E. coli* isolates were tested. All isolates were resistant to ATM, likely due to co-production of an AmpC-type β-lactamase and/or a class A extended-spectrum β-lactamase and/or PBP3 modifications (Table 1). Among the tested isolates, 70.8% (34/48) were resistant to ATM-AVI.

**Fig. 1:**
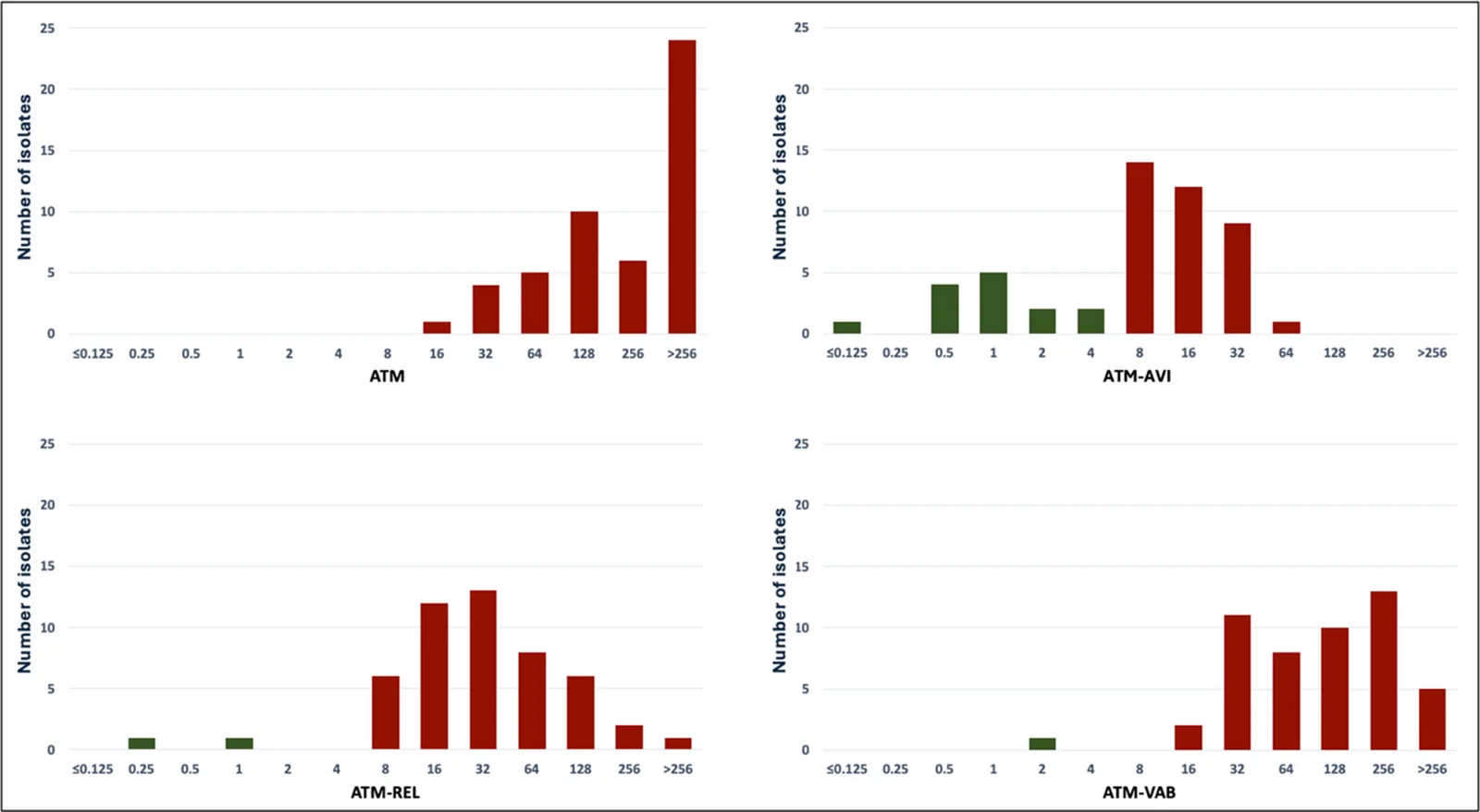
MICs distribution of aztreonam, aztreonam/avibactam, aztreonam/relebactam and aztreonam/vaborbactam (in mg/L). Red bars indicate resistant isolates, green bars indicate susceptible isolates, according to aztreonam breakpoint (≤ 4 mg/L). Abbreviations: ATM, aztreonam; ATM-AVI, aztreonam-avibactam; ATM-REL, aztreonam-relebactam; ATM-VAB, aztreonam-vaborbactam

Given the MICs distribution for each combination, ATM-REL appeared to be more effective than ATM-VAB, but both were markedly less effective than ATM-AVI (Fig. 1). MIC_50_ values were 8, 32, and 128 mg/L, and MIC_90_ values were 32, 128, and 256 mg/L, for ATM-AVI, ATM-REL and ATM-VAB, respectively.

Association with relebactam or vaborbactam showed poor susceptibility rates (4.2% (2/48) and 2.1% (1/48), respectively). Nevertheless, avibactam, relebactam, and vaborbactam decreased ATM MICs by ≥ 4 dilutions in 68.7% (33/48), 29.2% (14/48), and 8.3% (4/48) of isolates, respectively.

When focusing on ATM-AVI-resistant strains (34/48 isolates), relebactam and vaborbactam decreased ATM MICs by ≥ 4 dilutions in 14.7% (5/34) and 0% (0/34) of the strains, respectively, although not reaching the ATM susceptibility breakpoint.

When focusing on ATM-AVI-susceptible strains (14/48 isolates), relebactam and vaborbactam decreased ATM MICs by ≥ 4 dilutions in 64.2% (9/14) and 21.4% (3/14) of the strains, respectively, leading to a susceptibility rate of 14.3% (2/14) and 7.1% (1/14), respectively.

Resistome analysis of the 48 isolates showed that ATM-AVI-resistant strains typically harbored insertions in their PBP3, along with NDM (mostly NDM-5) and CMY β-lactamase genes (mostly CMY-42, and to a lesser extent other CMY-2 variants such as CMY-4 or CMY-145). For ATM-REL, 30/34 (88.2%) isolates with an ATM MIC decrease < 4-fold after relebactam addition harbored *bla*_CMY_ genes, compared to 8/14 (57.1%) with ≥ 4-fold decrease. None of the two ATM-REL-susceptible isolates harbored *bla*_CMY_. Aside from isolates harboring *bla*_CMY_ genes, all strains with ≥ 4-fold higher MICs for ATM-REL compared to ATM-AVI carried *bla*_NDM_ genes associated with *bla*_CTX-M-15_-like genes and PBP3 insertions, along with either *bla*_OXA-1_ or *bla*_TEM-1_ β-lactamase genes.

For ATM-VAB, 37/44 (84.1%) isolates with an ATM MIC decrease < 4-fold after vaborbactam addition harbored *bla*_CMY_ genes, compared to 1/4 (25%) with ≥ 4-fold decreases, and *bla*_CMY_ was not detected in the single ATM-VAB-susceptible isolate. This latter isolate also lacked *bla*_OXA_-type β-lactamase genes and PBP3 modifications.

## Discussion

This study evaluated the in vitro activity of ATM combined with three already-marketed BLIs against a collection of 48 well-characterized ATM-resistant MBL-producing *E. coli* isolates. Avibactam proved the most effective inhibitor for restoring aztreonam activity against MBL-producing *E. coli* isolates, in accordance with previous report [7–10]. The addition of REL, and to an even lesser extent VAB, was poorly effective in restoring susceptibility to ATM, with ≥ 4-fold MIC reduction in only 29.2% and 8.3% of isolates, respectively. Notably, neither REL nor VAB restored aztreonam susceptibility in any ATM-AVI-resistant isolate. This finding aligns with the limited data available to date: four ATM-AVI-resistant NDM-producing *E. coli* in the study by Emeraud et al., and one such isolate in the study by Vázquez-Ucha et al., were all non-susceptible to aztreonam combined with either relebactam or vaborbactam [9, 10]. For such ATM-AVI-resistant *E. coli*, combination with newer BLIs (e.g., zidebactam) should be considered [16].

In line with published data, ATM-AVI resistance in our collection of *E. coli* isolates was primarily mediated by the combination of PBP3 insertions, CMY-type β-lactamases, and NDM-type MBLs [2]. Within this resistance pattern, CMY-type enzymes seem to play a pivotal role in the failure of both relebactam and vaborbactam to restore aztreonam susceptibility. Indeed, CMY genes were less frequently detected among isolates where REL or VAB reduced ATM MICs by ≥ 4-fold, and were absent from the few isolates susceptible to ATM-REL and ATM-VAB combinations. This observation aligns with another study reporting the relatively poor inhibitory activity of vaborbactam against class C β-lactamases, as well as the lower inhibitory activity of relebactam compared to avibactam [17]. The few isolates lacking *bla*_CMY_ genes but showing significantly higher ATM-REL than ATM-AVI MICs harbored *bla*_CTX-M-15_ genes along with either *bla*_OXA-1_ or *bla*_TEM-1_ β-lactamase genes, again consistent with the superior inhibitory activity of avibactam over relebactam against these β-lactamases [6]. It is important to note that we did not assess the expression levels of plasmid-mediated β-lactamases or non-enzymatic resistance mechanisms. Therefore, their contribution to resistance to ATM-based combinations in these isolates remains unclear.

Our results contrast with previous studies (that also includes *Klebsiella pneumonaie* and *Citrobacter freundii* besides *E. coli*) showing that tripartite combinations of ATM with imipenem-relebactam or meropenem-vaborbactam demonstrated higher in vitro efficacy against MBL-producing Enterobacterales, which may explain successful reports of these regimens in treating MBL-producing *Enterobacterales* infections [11–13, 18]. In our study, ATM was tested in dual combination with BLIs, without any carbapenem addition. This discrepancy may stem from the associated carbapenem (i.e., imipenem or meropenem) retaining partial activity despite MBL production [19]. Indeed, carbapenems are not hydrolyzed by ESBLs or AmpCs co-produced by these strains, and are only slightly affected by PBP3 mutations, carbapenems (especially imipenem) binding most strongly to PBP2 whereas aztreonam only tightly bonds to PBP3 in *E. coli* [20]. Accordingly, our experimental design does not fully reflect the clinical scenario in which aztreonam would typically be added to existing combinations, which should be considered a limitation of our study.

In conclusion, ATM combined with relebactam or vaborbactam should not be recommended for treating infections caused by ATM-AVI-susceptible MBL-producing *E. coli*, given its potentially lower efficacy compared to ATM-AVI. The use of ATM-relebactam or ATM-vaborbactam would require detailed knowledge of the β-lactamase profile expressed by a given isolate, as well as susceptibility testing, which may not be feasible in real time. Imipenem-relebactam and meropenem-vaborbactam should rather be reserved for the treatment of infections caused by KPC-producing Enterobacterales, in accordance with current guidelines, as these BLIs were primarily designed to inhibit KPC carbapenemases. In ATM-AVI-resistant *E. coli*, ATM combined with relebactam or vaborbactam does not represent an effective in vitro alternative.

## Data Availability

The data that support the findings of this study are available on request from the corresponding author.
